# Negative evaluation fear and sharing avoidance on WeChat Moments: exhaustion and face

**DOI:** 10.3389/fpsyg.2026.1793555

**Published:** 2026-04-09

**Authors:** Yiqi Liu

**Affiliations:** Faculty of Humanities and Arts, Macau University of Science and Technology, Taipa, Macao SAR, China

**Keywords:** fear of negative evaluation, acquisitive face orientation, social network exhaustion, sharing avoidance, stressor–strain–outcome framework, WeChat Moments

## Abstract

**Introduction:**

Although existing social media research has predominantly focused on problematic use, such as overuse and addiction, considerably less attention has been paid to voluntary disengagement behaviors on socially embedded platforms. This study investigates the psychological mechanisms underlying social media sharing avoidance on WeChat Moments, a prominent Chinese social networking context characterized by strong interpersonal visibility.

**Methods:**

Drawing on the stressor–strain–outcome (SSO) framework, this study conceptualizes fear of negative evaluation as a stressor, social network exhaustion as a strain response, and sharing avoidance as a behavioral outcome. Data were collected in July 2024 from 353 active WeChat Moments users in China through an online questionnaire distributed via WeChat Moments using a snowball sampling strategy. The data were analyzed using partial least squares structural equation modeling (PLS-SEM).

**Results:**

The results show that fear of negative evaluation directly increases sharing avoidance and also indirectly influences avoidance through social network exhaustion, which serves as a partial mediator. Furthermore, acquisitive face orientation strengthens the effect of fear of negative evaluation on social network exhaustion and intensifies the mediated pathway toward sharing avoidance.

**Discussion:**

By integrating psychological stress theory with culturally grounded self-evaluative concerns, this study extends media psychology research on social media avoidance. The findings highlight how culturally embedded norms of social approval shape users’ emotional experiences and participation behaviors on social media.

## 1 Introduction

Social media platforms have become deeply embedded in everyday social interaction, yet users do not always respond to digital engagement with increased participation. In socially dense platforms such as WeChat Moments, where online audiences often overlap with offline social networks, users frequently remain present on the platform while reducing visible participation, particularly by limiting or avoiding content sharing. This phenomenon reflects a form of social media sharing avoidance, in which individuals strategically withdraw from active self-presentation while maintaining necessary social connections. Understanding why users adopt such avoidance behaviors is increasingly important for explaining participation dynamics in relationally embedded social media environments.

Existing research on social media avoidance has primarily emphasized privacy concerns, self-disclosure risks, social pressure, and information overload (Gibbs et al., 2010; Rui and Stefanone, 2013; Hollenbaugh and Ferris, 2014; Lee and Cho, 2018; Chen et al., 2019; Kamalou et al., 2019; Zhang et al., 2019; Zeng and Zhu, 2021). However, limited attention has been paid to evaluation-related psychological stress, particularly the role of fear of negative evaluation (*FONE*) in shaping sharing avoidance in socially visible social media environments. Prior research suggests that users who anticipate being widely observed and evaluated tend to become more cautious in online self-presentation, which may reduce their willingness to share content. For example, Zeng and Zhu (2021) found in the WeChat context that evaluation-related fears significantly reduced online self-disclosure. This issue is particularly salient in China, where WeChat serves as an extension of offline social relationships and where online sharing is closely tied to interpersonal visibility and social evaluation.

WeChat Moments provides a socially dense and visibility-sensitive environment in which online audiences often overlap with offline interpersonal networks. In such settings, content sharing is closely tied to self-presentation and social evaluation, making users particularly attentive to how they may be perceived by others. In China, WeChat functions not merely as a communication tool but as an infrastructure embedded in everyday social and professional life (Jiang, 2023). Because users often rely on WeChat to maintain interpersonal and work-related ties, they cannot easily withdraw from the platform even when they experience fatigue or stress. Instead, they may adopt partial disengagement strategies, such as browsing passively or reducing visible participation, which makes sharing avoidance a particularly meaningful behavioral outcome in this context.

Fear of negative evaluation has long been recognized as a core feature of social anxiety, reflecting individuals’ concerns about being negatively judged by others (Liu and Zhang, 2010; Watson and Friend, 1969; Weeks et al., 2008). In socially visible social media environments, content sharing often involves anticipated scrutiny from acquaintances, colleagues, and friends, which may heighten users’ sensitivity to potential negative judgments. Such evaluation-related pressure can increase the emotional and cognitive demands of self-presentation, gradually depleting users’ psychological resources and resulting in social network exhaustion. When users experience this form of exhaustion, they may become more cautious about visible participation and increasingly avoid sharing content in order to reduce further evaluative exposure.

The strength of this process may vary depending on culturally embedded self-evaluative concerns. In Chinese cultural contexts, face plays an important role in shaping individuals’ social behavior and sensitivity to others’ judgments (Zhao, 2012; Li and Song, 2004; Wang and Yang, 2005). Acquisitive face orientation (*AFO*) reflects individuals’ motivation to gain social approval and enhance their public image through positive evaluations from others (Zhang et al., 2011; Chou, 1996). Individuals with stronger acquisitive face orientation are likely to invest greater psychological resources in impression management and the maintenance of a favorable social image. From the perspective of conservation of resources theory, such heightened investment does not buffer the stress associated with evaluation; rather, it makes evaluation-related pressure more resource-consuming because more cognitive and emotional resources are at stake once one’s social image is perceived as vulnerable (Hobfoll, 1989). In addition, emotional labor theory suggests that maintaining a socially desirable image requires continuous regulation of self-presentation and emotional display (Hochschild, 1983). Accordingly, *AFO* is expected to intensify, rather than alleviate, the effect of *FONE* on *SNE.*

To systematically analyze these relationships, this study draws on the stressor–strain–outcome (SSO) model originally proposed by Koeske and Koeske (1993). The SSO framework explains how psychological stressors generate strain responses that subsequently lead to behavioral outcomes. In the present study, fear of negative evaluation (*FONE*) is conceptualized as the psychological stressor, social network exhaustion (*SNE*) as the strain response, and social media sharing avoidance behavior (*SMSAB*) as the behavioral outcome. In addition, acquisitive face orientation (*AFO*) is introduced as a culturally grounded moderator that may shape the extent to which evaluation-related stress translates into social network exhaustion. By applying the SSO framework to the context of WeChat Moments, this study seeks to clarify the psychological mechanism underlying sharing avoidance in socially embedded digital environments and to highlight the role of culturally embedded self-evaluative concerns in shaping users’ participation behaviors.

By integrating fear of negative evaluation into the stressor–strain–outcome framework, this study extends the SSO model to evaluation-related psychological stress in socially embedded social media environments. Rather than treating reduced participation simply as platform disengagement, the present study conceptualizes social media sharing avoidance as an adaptive behavioral response to evaluation pressure. In addition, by incorporating acquisitive face orientation into the model, this study highlights how culturally embedded self-evaluative concerns may intensify the translation of psychological stress into emotional exhaustion. In this way, the study not only clarifies the psychological mechanism underlying sharing avoidance on WeChat Moments, but also contributes to a more culturally grounded understanding of digital participation and withdrawal in Chinese social media contexts.

**Figure 1:**
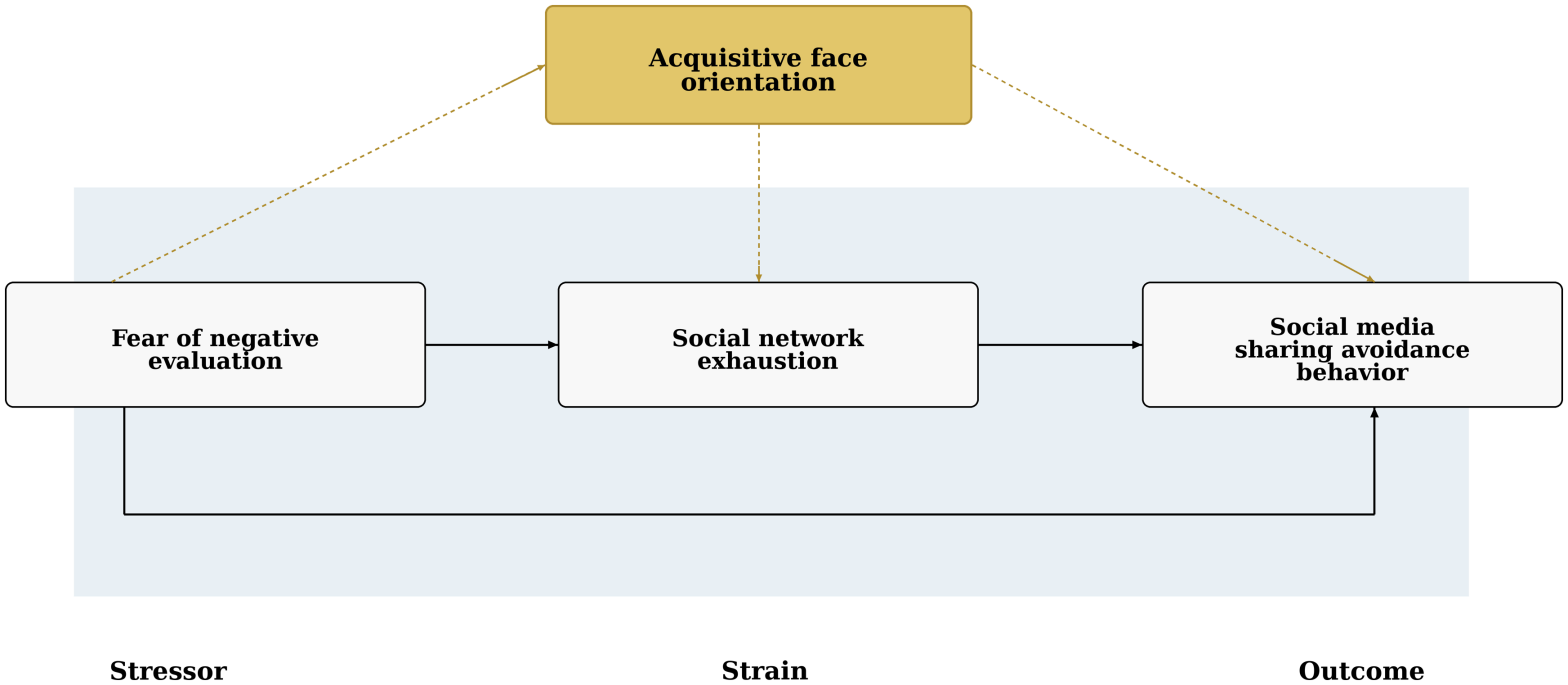
Conceptual model of the study.

Based on this theoretical framework, the conceptual model proposed in Figure 1 examines the relationships among fear of negative evaluation (*FONE*), social network exhaustion (*SNE*), acquisitive face orientation (*AFO*), and social media sharing avoidance behavior (*SMSAB*). Accordingly, the following hypotheses are proposed:

*H1*: Fear of negative evaluation (*FONE*) is positively associated with social media sharing avoidance behavior (*SMSAB*).

*H2*: Social network exhaustion (*SNE*) mediates the positive relationship between fear of negative evaluation (*FONE*) and social media sharing avoidance behavior (*SMSAB*).

*H3*: Acquisitive face orientation (*AFO*) moderates the relationship between fear of negative evaluation (*FONE*) and social network exhaustion (*SNE*), such that the positive relationship is stronger at higher levels of acquisitive face orientation.

*H4*: Acquisitive face orientation (*AFO*) moderates the indirect relationship between fear of negative evaluation (*FONE*) and social media sharing avoidance behavior (*SMSAB*) via social network exhaustion (*SNE*), such that the indirect effect is stronger at higher levels of acquisitive face orientation.

To empirically examine the proposed model, this study adopts a quantitative research design. The following section describes the sample, measurement instruments, and analytical procedures used to test the proposed direct, mediating, and moderated relationships.

## 2 Methods and materials

To empirically examine the proposed model, this study employed a quantitative research design. The following subsections describe the sample, measurement instruments, and analytical procedures.

### 2.1 Sample

The study was designed as a cross-sectional online survey conducted among WeChat users in China. The study employed a non-probability snowball sampling strategy using WeChat-based network diffusion. The questionnaire link was initially distributed through the researcher’s WeChat Moments, and respondents were invited to further circulate the survey within their own social networks on WeChat. Participation was entirely voluntary, and no monetary or material compensation was provided for completing the survey. Upon accessing the survey link, participants were presented with an introductory page explaining the general purpose of the study, the approximate duration of participation, and assurances regarding anonymity and confidentiality. Participants were informed that all responses would be used solely for academic research purposes. Only those who provided explicit informed consent were able to proceed to the questionnaire.

To ensure data quality, an attention check item was embedded in the survey. Responses that failed this check were excluded from further analysis. After data screening, a total of 353 valid responses were retained for subsequent statistical analyses. All questionnaire items were set as mandatory, such that only fully completed surveys were recorded as valid submissions. As shown in Table 1, the sample comprised 65.7% male and 34.3% female respondents. In terms of age, participants were concentrated primarily in the 21–30 age group (53.0%), followed by the 51–60 group (20.1%) and the 31–40 group (16.1%). Most respondents held a bachelor’s degree (57.2%), while 32.3% had completed high school or vocational secondary school and 9.9% had attained a postgraduate degree or above.

**Table 1:** Sociodemographic characteristics.

Variables	Category	*n*	%
Gender	Male	232	65.7
	Female	121	34.3
Age	0–20 years	6	1.7
	21–30 years	187	53.0
	31–40 years	57	16.1
	41–50 years	9	2.5
	51–60 years	71	20.1
	61 years and above	23	6.5
Educational level	Junior high school or below	2	0.6
	High school/vocational secondary school	114	32.3
	Bachelor’s degree	202	57.2
	Postgraduate degree or above	35	9.9

Percentages may not sum to 100 due to rounding.

### 2.2 Measuring instruments

All study variables were measured using multi-item scales on a five-point Likert scale ranging from 1 (“strongly disagree”) to 5 (“strongly agree”). The measurement instruments were adapted from established and validated scales used in prior research to ensure content validity and theoretical consistency. The sources of the items for each construct are reported in Table 2.

- Fear of Negative Evaluation (*FONE*) was measured using an 8-item Chinese version adapted from Jiang (2023), which was based on the straightforward items of the Brief Fear of Negative Evaluation Scale (BFNE-S; Leary, 1983; Weeks et al., 2005). The scale assesses individuals’ concerns and apprehension about being negatively judged or evaluated by others. Following Jiang (2023), only the 8 straightforward items were retained, because prior research has suggested that the reverse-worded items in the original 12-item version may introduce confusion and measurement error.
- Social Network Exhaustion (*SNE*) was measured using a 6-item scale directly adapted from Pradhan (2022), without further shortening. This scale captures users’ subjective feelings of psychological fatigue, emotional depletion, and cognitive overload resulting from sustained engagement with social networking platforms. The construct reflects the strain component in the stressors–strains–outcomes framework and has been validated in prior research on social media fatigue and discontinuance behaviors.
- Acquisitive Face Orientation (*AFO*) was measured using a 6-item scale adapted from Wang et al. (2016), without further shortening. This construct reflects individuals’ motivation to gain social approval, enhance social image, and obtain positive evaluations from others. The scale emphasizes culturally embedded self-evaluative concerns that are particularly salient in Chinese social interaction contexts and has been widely employed in research on face consciousness and online self-presentation.
- Social Media Sharing Avoidance Behavior (*SMSAB*) was measured using an 8-item scale directly adapted from Jiang (2023), without further shortening. This scale assesses the extent to which users deliberately reduce, restrict, or avoid sharing content on social media platforms as a strategic behavioral response to psychological pressure or social discomfort.

**Table 2:** Constructs and items.

Construct	Items	Source
Fear of Negative Evaluation (*FONE*)	*FONE1*: I worry about what other people think of me, even though I know their opinions will not change me. *FONE2*: I often fear that others will notice my shortcomings. *FONE3*: I worry that others will not approve of me. *FONE4*: I am afraid that people will deliberately find fault with me. *FONE5*: When I talk to other people, I worry a lot about what they think of me. *FONE6*: I often worry about the impression I make on others. *FONE7*: Sometimes I think I care too much about what other people think of me. *FONE8*: I often worry that I will say or do the wrong thing.	Jiang (2023)
Social Network Exhaustion (*SNE*)	*SNE1*: I feel tired when using WeChat. *SNE2*: I feel bored when using WeChat. *SNE3*: I feel drained from using WeChat. *SNE4*: I feel worn out from using WeChat. *SNE5*: I feel disinterested in whether there are new things happening on WeChat. *SNE6*: The notifications or alerts of new postings on WeChat do not really affect me.	Pradhan (2022)
Acquisitive Face Orientation (*AFO*)	*AFO1*: I hope people think that I can do better than most others. *AFO2*: I am happy to show my bright side. *AFO3*: I hope that, in the eyes of others, I have a better life than most people. *AFO4*: Praise and admiration are important to me. *AFO5*: I like to talk about new topics. *AFO6*: I will seize any opportunities to show myself off to gain face.	Wang et al. (2016)
Social Media Sharing Avoidance Behavior (*SMSAB*)	*SMSAB1*: I do not post updates about my daily life on WeChat Moments. *SMSAB2*: I do not express my emotional state on WeChat Moments. *SMSAB3*: I do not post selfies on WeChat Moments. *SMSAB4*: I do not share my location on WeChat Moments. *SMSAB5*: I do not post amusing things I have seen on WeChat Moments. *SMSAB6*: I do not post important things that I am familiar with on WeChat Moments. *SMSAB7*: I do not share or comment on products on WeChat Moments. *SMSAB8*: I do not repost content and add comments on WeChat Moments.	Jiang (2023)

To ensure linguistic equivalence and cultural appropriateness, all measurement items were translated into Chinese and then back-translated into English following the standard back-translation procedure proposed by Brislin (1970). Discrepancies between the original and back-translated versions were discussed and resolved to achieve semantic consistency. This process helped enhance the reliability and validity of the instruments in the Chinese research context.

## 3 Results

This section presents the empirical results of the study. We first assess the measurement model, followed by the structural model and the tests of the direct, mediating, moderating, and moderated mediation effects. Partial least squares structural equation modeling (PLS-SEM) was employed to test the proposed conceptual model. Consistent with established guidelines (Hair et al., 2019), the analysis proceeded in two stages: assessment of the measurement model and evaluation of the structural model.

### 3.1 Measurement model

The measurement model was assessed in terms of indicator reliability, internal consistency reliability, convergent validity, and discriminant validity. As shown in Table 3, the standardized outer loadings ranged from 0.640 to 0.874, indicating acceptable item reliability overall. Specifically, the item loadings ranged from 0.640 to 0.837 for *AFO*, 0.655 to 0.816 for fear of negative evaluation *FONE,* 0.788 to 0.847 for *SMSAB*, and 0.766 to 0.874 for *SNE*.

**Table 3:** Measurement model assessment.

Construct	Item	Outer loading	Cronbach’s alpha	rho_A	CR	AVE
*AFO*	*AFO1*	0.802	0.875	0.903	0.904	0.613
	*AFO2*	0.640				
	*AFO3*	0.797				
	*AFO4*	0.813				
	*AFO5*	0.794				
	*AFO6*	0.837				
*FONE*	*FONE1*	0.655	0.903	0.907	0.922	0.596
	*FONE2*	0.794				
	*FONE3*	0.793				
	*FONE4*	0.763				
	*FONE5*	0.791				
	*FONE6*	0.816				
	*FONE7*	0.787				
	*FONE8*	0.766				
*SMSAB*	*SMSAB1*	0.843	0.935	0.936	0.946	0.686
	*SMSAB2*	0.821				
	*SMSAB3*	0.823				
	*SMSAB4*	0.839				
	*SMSAB5*	0.840				
	*SMSAB6*	0.824				
	*SMSAB7*	0.847				
	*SMSAB8*	0.788				
*SNE*	*SNE1*	0.859	0.915	0.917	0.934	0.703
	*SNE2*	0.817				
	*SNE3*	0.863				
	*SNE4*	0.874				
	*SNE5*	0.848				
	*SNE6*	0.766				

Internal consistency reliability was satisfactory for all constructs. Cronbach’s alpha values ranged from 0.875 to 0.935, rho_A values ranged from 0.903 to 0.936, and composite reliability (CR) values ranged from 0.904 to 0.946, all exceeding the recommended threshold of 0.70. Convergent validity was also supported, as the average variance extracted (AVE) values ranged from 0.596 to 0.703, all above the recommended cutoff of 0.50. Specifically, the AVE values were 0.613 for *AFO*, 0.596 for *FONE*, 0.686 for *SMSAB*, and 0.703 for *SNE*. Collinearity diagnostics for the outer model also indicated no serious multicollinearity concerns, with indicator-level VIF values ranging from 1.000 to 3.323.

Discriminant validity was examined using both the Fornell–Larcker criterion and the heterotrait–monotrait ratio (HTMT). As shown in Table 4, the square roots of the AVE for each construct were greater than the corresponding inter-construct correlations. In addition, all HTMT values were below the conservative threshold of 0.85, ranging from 0.417 to 0.781 (see Table 5), indicating satisfactory discriminant validity among the constructs.

**Table 4:** Fornell–Larcker criterion.

Construct	*AFO*	*FONE*	*SMSAB*	*SNE*
*AFO*	0.783			
*FONE*	0.378	0.772		
*SMSAB*	0.423	0.612	0.828	
*SNE*	0.489	0.616	0.724	0.839

**Table 5:** Heterotrait–monotrait ratio (HTMT).

Construct	*AFO*	*FONE*	*SMSAB*	*SNE*
*AFO*				
*FONE*	0.417			
*SMSAB*	0.452	0.658		
*SNE*	0.513	0.672	0.781	

All HTMT values were below the conservative threshold of 0.85, indicating satisfactory discriminant validity.

Taken together, these results indicate that the measurement model demonstrates adequate reliability and validity.

### 3.2 Structural model

The structural model was evaluated in terms of collinearity, model fit, explanatory power, predictive relevance, and effect size (Table 6). Collinearity diagnostics indicated that the inner-model VIF values ranged from 1.104 to 1.612, all below the commonly recommended threshold, suggesting that multicollinearity was not a serious concern. Model fit was assessed using the standardized root mean square residual (SRMR). The estimated model yielded an SRMR value of 0.080 (saturated model = 0.079), indicating an acceptable level of model fit according to commonly used PLS-SEM criteria.

**Table 6:** Structural model evaluation.

Criterion	Construct/path	Value
Inner-model VIF	*AFO* → *SNE*	1.167
	*FONE* → *SMSAB*	1.612
	*FONE* → *SNE*	1.275
	*SNE* → *SMSAB*	1.612
	*AFO* × *FONE* → *SNE*	1.104
SRMR	Saturated model	0.079
	Estimated model	0.080
*R* ^2^	*SNE*	0.496
Adjusted *R*^2^	*SNE*	0.492
*Q* ^2^	*SNE*	0.342
*R* ^2^	*SMSAB*	0.569
Adjusted *R*^2^	*SMSAB*	0.567
*Q* ^2^	*SMSAB*	0.383
*f* ^2^	*FONE* → *SNE*	0.504
	*FONE* → *SMSAB*	0.103
	*SNE* → *SMSAB*	0.451
	*AFO* → *SNE*	0.149
	*AFO* × *FONE* → *SNE*	0.079

SRMR, standardized root mean square residual; *R*^2^, coefficient of determination; *Q*^2^, cross-validated redundancy; *f*^2^, effect size; VIF, variance inflation factor.

In terms of explanatory power, the model explained 49.6% of the variance in *SNE* (*R*^2^ = 0.496; adjusted *R*^2^ = 0.492) and 56.9% of the variance *SMSAB* (*R*^2^ = 0.569; adjusted *R*^2^ = 0.567), indicating moderate to substantial explanatory power. Predictive relevance was assessed using the blindfolding procedure. The *Q*^2^ values for the endogenous constructs were 0.342 for *SNE* and 0.383 for *SMSAB*, both above zero, indicating satisfactory predictive relevance of the model.

The effect size estimates (*f*^2^) further showed that *FONE* had a large effect on *SNE* (*f*^2^ = 0.504) and a small-to-moderate direct effect on *SMSAB* (*f*^2^ = 0.103). *SNE* had a large effect on *SMSAB* (*f*^2^ = 0.451). In addition, *AFO* had a small-to-moderate effect on *SNE* (*f*^2^ = 0.149), while the interaction term (*AFO × FONE*) exerted a small effect on *SNE* (*f*^2^ = 0.079). These findings suggest that the primary explanatory force of the model lies in the paths from FONE to SNE and from *SNE* to *SMSAB*, while the moderating effect of *AFO* is meaningful but comparatively smaller.

**Figure 2:**
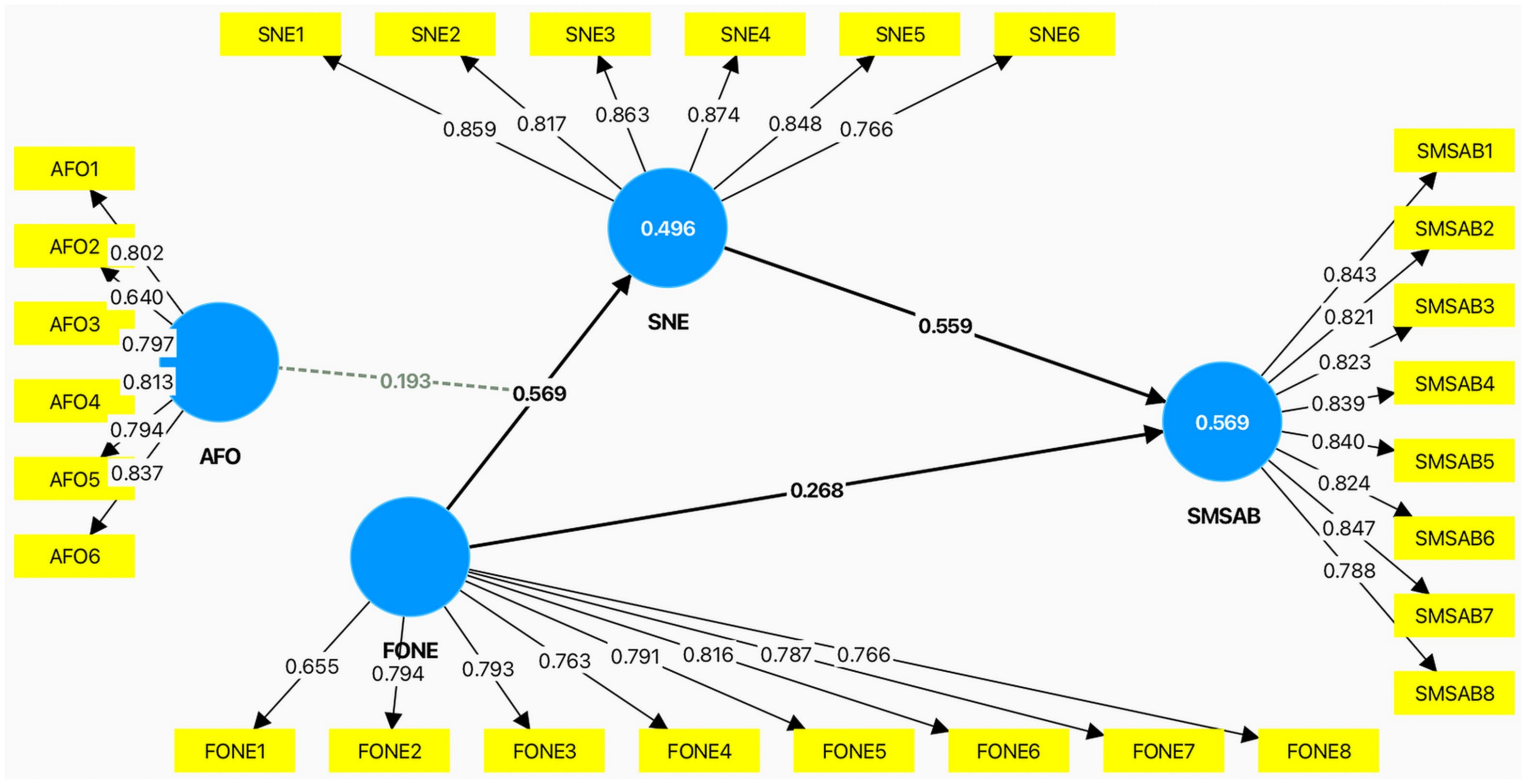
Structural model with standardized path coefficients and indicator loadings.

Overall, the structural model demonstrates satisfactory performance in terms of explained variance, supporting its suitability for subsequent hypothesis testing. Figure 2 presents the main structural model with standardized path coefficients, indicator loadings, and *R*^2^ values. The following sections therefore proceed to examine the specific hypothesized relationships, including direct effects, mediation, moderation, and moderated mediation effects, in greater detail.

### 3.3 Direct effects analysis

As shown in Table 7, the bootstrapping results obtained from SmartPLS indicate that *FONE* has a positive and significant direct association with *SMSAB* (*β* = 0.268, SE = 0.089, *t* = 3.000, *p* = 0.003) and a stronger positive association with *SNE* (*β* = 0.569, SE = 0.054, *t* = 10.480, *p* < 0.001). In turn, *SNE* is positively and significantly associated with *SMSAB* (*β* = 0.559, SE = 0.081, *t* = 6.905, *p* < 0.001). These direct paths suggest that individuals who experience greater fear of negative evaluation are more likely to feel exhausted in social networking environments and, correspondingly, more likely to avoid visible content sharing.

**Table 7:** Direct effects.

Path	*β*	SE	*t*	*p*
*FONE* → *SMSAB*	0.268	0.089	3.000	0.003
*FONE* → *SNE*	0.569	0.054	10.480	< 0.001
*SNE* → *SMSAB*	0.559	0.081	6.905	< 0.001

### 3.4 Mediation effects analysis

The indirect effect of *FONE* on *SMSAB* via *SNE* was significant (*β* = 0.318, SE = 0.057, *t* = 5.569, *p* < 0.001, 95% CI [0.215, 0.437]), indicating a meaningful mediating pathway from evaluation-related stress to sharing avoidance through social network exhaustion. Because the direct effect of *FONE* on *SMSAB* remained significant after including *SNE* in the model (*β* = 0.268, SE = 0.089, *t* = 3.000, *p* = 0.003, 95% CI [0.090, 0.443]), the mediation pattern is consistent with partial mediation. These findings support the view that social network exhaustion serves as a key strain mechanism through which fear of negative evaluation is translated into social media sharing avoidance (see Table 8).

**Table 8:** Mediation analysis.

Effect type	Path	*β*	SE	*t*	*p*	95% CI
Direct effect	*FONE* → *SMSAB*	0.268	0.089	3.000	0.003	[0.090, 0.443]
Indirect effect	*FONE* → *SNE* → *SMSAB*	0.318	0.057	5.569	< 0.001	[0.215, 0.437]
Partial mediation	*FONE* → *SNE* → *SMSAB*					

### 3.5 Moderation effects analysis

The moderating effect of acquisitive face orientation (*AFO*) was examined by testing the interaction term between *AFO* and fear of negative evaluation (*FONE*) in predicting social network exhaustion (*SNE*). As shown in Table 9, the interaction effect was positive and statistically significant (*β* = 0.193, SE = 0.045, *t* = 4.274, *p* < 0.001, 95% CI [0.108, 0.288]), indicating that the positive relationship between *FONE* and *SNE* becomes stronger as *AFO* increases.

**Table 9:** Moderation and moderated mediation results.

Effect type	Path	*β*	SE	*t*	*p*	95% CI
Direct effect	*AFO* → *SNE*	0.295	0.047	6.319	*< 0.001*	[0.205, 0.385]
Interaction effect	*AFO* × *FONE* → *SNE*	0.193	0.045	4.274	*< 0.001*	[0.108, 0.288]
Simple slope (low *AFO*)	*FONE* → *SNE*	0.376				
Simple slope (high *AFO*)	*FONE* → *SNE*	0.762				
Conditional indirect effect	*AFO* × *FONE* → *SNE* → *SMSAB*	0.108	0.030	3.600	*< 0.001*	[0.056, 0.173]
Indirect effect	*AFO* → *SNE* → *SMSAB*	0.165	0.037	4.525	*< 0.001*	[0.101, 0.243]

**Figure 3:**
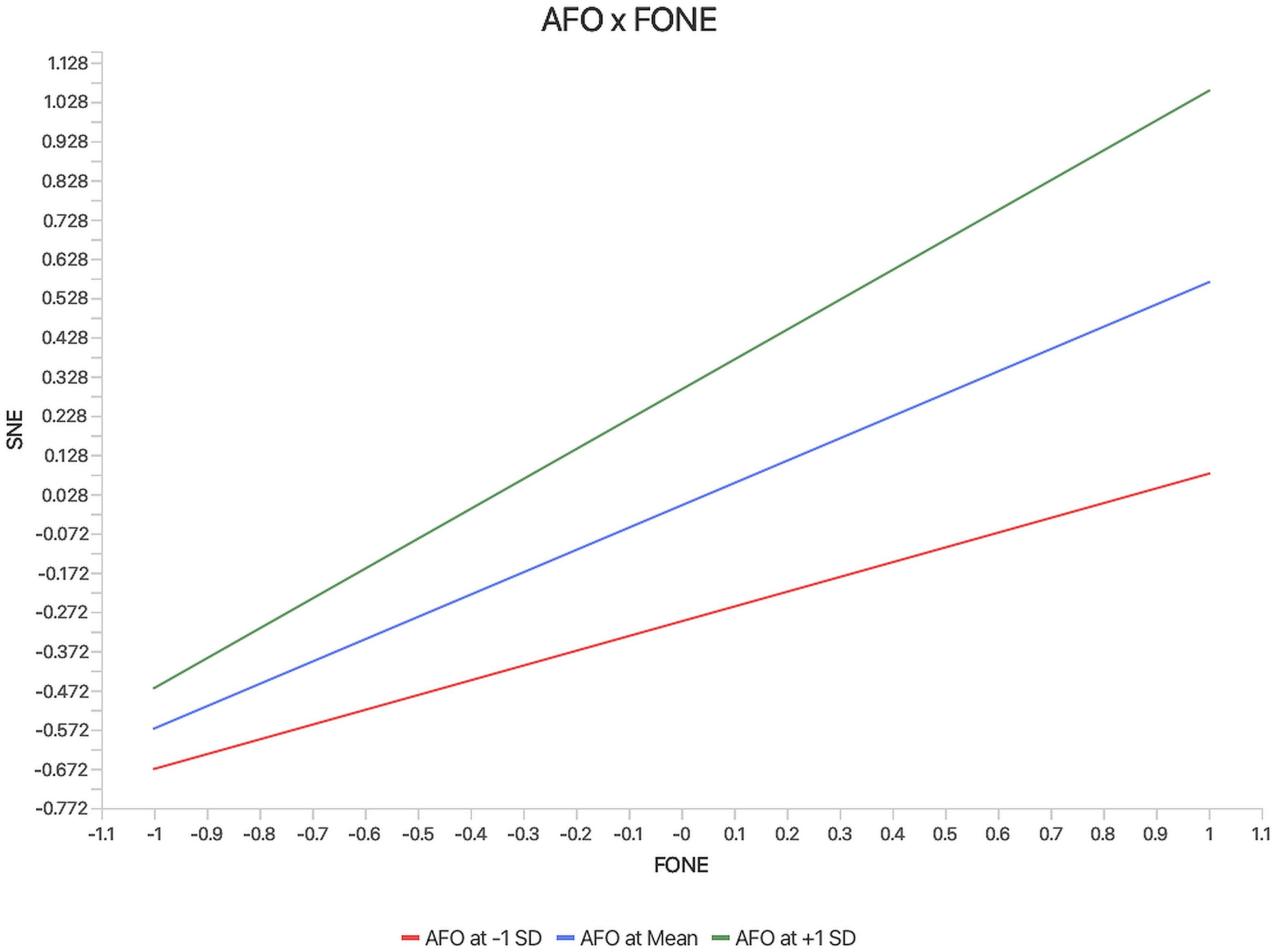
Simple slope plot of the moderating effect of *AFO* on the relationship between *FONE* and *SNE.*

In addition to the interaction effect, *AFO* also had a significant positive direct association with *SNE* (*β* = 0.295, SE = 0.047, *t* = 6.319, *p* < 0.001, 95% CI [0.205, 0.385]), suggesting that face-related self-evaluative concerns contribute to the strain response both directly and by intensifying the influence of *FONE*. Simple slope probing further showed that the effect of *FONE* on *SNE* was stronger at higher levels of *AFO* (*β* = 0.762) than at lower levels of *AFO* (*β* = 0.376), which is consistent with the moderating pattern illustrated in Figure 3. These results support the view that acquisitive face orientation amplifies the psychological strain associated with evaluation-related stress.

### 3.6 Moderated mediation effects analysis

The SmartPLS results further indicate a moderated mediation pattern. As shown in Table 9, the specific indirect effect of the interaction term (AFO × FONE) on social media sharing avoidance behavior (SMSAB) through SNE was significant (*β* = 0.108, SE = 0.030, t = 3.600, *p < 0.001*, 95% CI [0.056, 0.173]), suggesting that the indirect effect of FONE on SMSAB via SNE varies as a function of AFO. In addition, the indirect effect of AFO on SMSAB through SNE was also significant (*β* = 0.165, SE = 0.037, *t* = 4.525, *p < 0.001*, 95% CI [0.101, 0.243]).

Taken together, these findings indicate that the indirect path from *FONE* to *SMSAB* through *SNE* is stronger when *AFO* is higher, which is consistent with the theorized moderated mediation mechanism. This pattern suggests that *AFO* strengthens the stress–strain relationship, thereby intensifying the indirect effect of *FONE* on *SMSAB* through *SNE*.

### 3.7 Summary of hypothesis testing results

Overall, the empirical results supported all proposed hypotheses. A summary of the hypothesis-testing results is presented in Table 10. Fear of negative evaluation (*FONE*) was positively associated with social media sharing avoidance behavior (*SMSAB*), supporting H1. Social network exhaustion (*SNE*) served as a significant partial mediator in the relationship between *FONE* and *SMSAB*, supporting H2. In addition, acquisitive face orientation (*AFO*) significantly moderated the relationship between *FONE* and *SNE*, such that the positive association became stronger at higher levels of *AFO*, supporting H3. Finally, the indirect effect of *FONE* on *SMSAB* through *SNE* was stronger when *AFO* was higher, supporting H4. Taken together, these findings provide full empirical support for the proposed conceptual model.

**Table 10:** Summary of hypothesis testing results.

Hypothesis	Statement	Result
H1	*FONE* is positively associated with *SMSAB*.	Supported
H2	*SNE* mediates the positive relationship between *FONE* and *SMSAB*.	Supported
H3	*AFO* moderates the relationship between *FONE* and *SNE*, such that the positive relationship is stronger at higher levels of *AFO*.	Supported
H4	*AFO* moderates the indirect relationship between *FONE* and *SMSAB* through *SNE*, such that the indirect effect is stronger at higher levels of *AFO*.	Supported

## 4 Discussion

### 4.1 Theoretical implications

The findings of this study provide important insights into the psychological processes associated with social media sharing avoidance behavior, highlighting the role of fear of negative evaluation (*FONE*) and clarifying how emotional strain and culturally embedded self-evaluative concerns are linked to users’ behavioral responses. Although fear of evaluation has been extensively examined in the psychology literature as a core component of social anxiety (Watson and Friend, 1969; Weeks et al., 2008; Liu and Zhang, 2010; Peng et al., 2019), it has received comparatively limited attention in communication and media studies. By incorporating *FONE* into the analysis of social media use, this study extends prior research and underscores its relevance for understanding users’ cognitive and behavioral responses in digitally mediated social contexts.

First, the results indicate that fear of negative evaluation is positively associated with social media sharing avoidance behavior. Previous studies have primarily attributed sharing avoidance to concerns about privacy, self-disclosure risks, or uncertainty reduction strategies (Gibbs et al., 2010; Rui and Stefanone, 2013; Hollenbaugh and Ferris, 2014). More recent research has also emphasized platform-specific affordances and social pressures as antecedents of avoidance behavior (Lee and Cho, 2018; Zhang et al., 2019). The present findings complement this literature by identifying evaluation-related anxiety as an important psychological correlate of sharing avoidance. This pattern is also consistent with prior research showing that fear of negative evaluation is associated with maladaptive online outcomes, including social anxiety and excessive internet use (Peng et al., 2019; Peng et al., 2020). This result is also consistent with Jiang’s (2023) study on Chinese social media users, which highlighted fear-related psychological factors as relevant to sharing avoidance, particularly in socially embedded platforms such as WeChat Moments.

Second, the mediating role of social network exhaustion provides empirical support for the stressor–strain–outcome (SSO) framework in explaining social media sharing avoidance behavior. Consistent with prior applications of the SSO model in digital media research (Zheng and Ling, 2021; Ou et al., 2023; Zhang et al., 2024), the results suggest that fear of negative evaluation functions as a psychological stressor associated with emotional and cognitive strain in the form of social network exhaustion. This exhaustion, in turn, is positively associated with users’ likelihood of avoiding sharing behaviors. Similar mechanisms have been observed in studies linking social media fatigue to use reduction and discontinuance behaviors (Bright et al., 2015; Bright and Logan, 2018; Fu et al., 2020; Gan et al., 2023). By providing empirical support for this mediating pathway, the present study clarifies how evaluation-related stress may be translated into avoidance behavior through accumulated emotional fatigue.

Third, the moderating effect of acquisitive face orientation underscores the importance of culturally embedded self-concepts in shaping users’ psychological responses to social media use. Prior research has shown that face orientation plays a critical role in self-presentation, impression management, and information sharing behaviors in Chinese cultural contexts (Chou, 1996; Zhang et al., 2011; Wang et al., 2016; Kumar et al., 2021). The present findings extend this literature by showing that acquisitive face orientation functions as a culturally grounded boundary condition that intensifies the relationship between fear of negative evaluation and social network exhaustion. In other words, individuals with higher acquisitive face orientation appear more vulnerable to evaluation-related stress in socially dense digital environments, where concerns about approval and image maintenance are especially salient.

Fourth, the moderated mediation results further extend the model by showing that acquisitive face orientation strengthens the indirect relationship between fear of negative evaluation and social media sharing avoidance through social network exhaustion. This pattern suggests that individuals high in acquisitive face orientation may face greater psychological costs in socially visible online environments, as stronger concerns about social image are associated with heightened evaluation-related stress and greater emotional exhaustion. This finding aligns with research emphasizing the cumulative psychological costs of impression management in socially dense networks (Maier et al., 2015) and further highlights how culturally embedded face concerns intensify evaluation-related stress processes in digital communication settings.

Taken together, this study makes three theoretical contributions. First, it extends the stressor–strain–outcome framework by moving beyond information- or technology-related stressors and demonstrating the relevance of evaluation-related psychological stress in explaining social media behavior. Second, it contributes to media psychology by linking fear of negative evaluation to a specific form of behavioral adjustment—social media sharing avoidance—and by framing this behavior as an adaptive response to evaluative pressure rather than merely a form of disengagement. Third, it contributes to cultural communication research by identifying acquisitive face orientation as a culturally grounded boundary condition that intensifies evaluation-related stress processes in socially dense digital environments.

### 4.2 Practical implications

From a practical perspective, the findings suggest that platform designers and managers should pay greater attention to the psychological burden created by socially visible online environments. Because fear of negative evaluation is positively associated with both social network exhaustion and sharing avoidance, platforms may benefit from reducing evaluative pressure through more flexible and user-centered design features. For example, customizable privacy settings, selective audience controls, or options that reduce the salience of public feedback may help users manage visibility and lower the psychological costs of participation.

These findings also suggest that reducing visible evaluative pressure may be especially important in relationally embedded platforms such as WeChat Moments, where online participation is closely tied to offline social relationships. More adaptive design strategies may help users remain socially connected without experiencing excessive exhaustion or withdrawing from visible participation.

### 4.3 Limitations and future directions

Despite the contributions of this study, several limitations should be acknowledged and addressed in future research. First, the study relied on self-reported data collected through an online survey, which may be subject to common method bias and social desirability effects. Respondents’ perceptions and reported behaviors may either overestimate or underestimate the strength of the proposed relationships. Future studies could incorporate behavioral data, digital trace measures, or mixed-method approaches to enhance measurement validity.

Second, the use of a cross-sectional research design limits the ability to draw causal inferences among the examined variables. Although the findings are theoretically grounded in the stressors–strains–outcomes framework, longitudinal or experimental designs would allow future research to test the proposed mechanisms more rigorously and to assess causal relationships more directly. For example, experimental studies could manipulate evaluative cues, audience visibility, or feedback conditions in simulated social media environments to examine whether stronger evaluation-related cues increase social network exhaustion and sharing avoidance. Such designs would also allow researchers to test whether individuals high in acquisitive face orientation are more sensitive to these manipulations, thereby providing stronger causal evidence for its boundary role in evaluation-related stress processes.

Third, social media sharing avoidance on WeChat Moments manifests in multiple forms, such as restricting post visibility to a limited time frame, reducing the frequency of original content posting, or selectively managing audience groups through blocking or customized visibility settings. In the present study, sharing avoidance was measured as a general construct without differentiating among these specific behavioral strategies. Future research could adopt more fine-grained classifications to examine whether distinct forms of sharing avoidance are driven by different psychological mechanisms.

Finally, given the culturally specific context of this study, the analysis focused on acquisitive face orientation as a key moderating factor. While face orientation captures an important dimension of self-evaluative concern in Chinese society, other culturally embedded constructs—such as guanxi or relational obligation—may further shape users’ responses to evaluation-related stress. Incorporating additional cultural variables in future studies would provide a more comprehensive understanding of social media avoidance behaviors in collectivist contexts.

## 5 Conclusion

This study provides empirical evidence that fear of negative evaluation is positively associated with social media sharing avoidance behavior and that this relationship is partially mediated by social network exhaustion. The findings further suggest that acquisitive face orientation functions as a culturally grounded boundary condition that intensifies evaluation-related stress processes in the socially dense environment of WeChat Moments. By applying the stressor–strain–outcome framework to evaluation-related psychological stress in a Chinese social media context, this study extends the SSO framework, contributes to media psychology research on social media avoidance, and adds to cultural communication research by highlighting the role of face-related concerns in shaping users’ behavioral responses. Taken together, the results underscore the importance of addressing evaluation-related stress and culturally embedded self-evaluative concerns when seeking to understand and support healthier patterns of participation in socially dense social media environments.

## Data Availability

The anonymized raw data supporting the conclusions of this article will be made available by the authors upon reasonable request.
